# Generating open-source 3D phytoplankton models by integrating photogrammetry with scanning electron microscopy

**DOI:** 10.3389/fmicb.2024.1429179

**Published:** 2024-07-15

**Authors:** Xuerong Sun, Robert J. W. Brewin, Christian Hacker, Johannes J. Viljoen, Mengyu Li

**Affiliations:** ^1^Centre for Geography and Environmental Science, Department of Earth and Environmental Science, Faculty of Environment, Science and Economy, University of Exeter, Cornwall, United Kingdom; ^2^Bioimaging Centre, University of Exeter, Exeter, United Kingdom; ^3^State Key Laboratory of Estuarine and Coastal Research, East China Normal University, Shanghai, China

**Keywords:** marine phytoplankton, scanning electron microscope, photogrammetry, 3D modeling, 3D printing, 3D model, open source

## Abstract

The community structure and ecological function of marine ecosystems are critically dependent on phytoplankton. However, our understanding of phytoplankton is limited due to the lack of detailed information on their morphology. To address this gap, we developed a framework that combines scanning electron microscopy (SEM) with photogrammetry to create realistic 3D (three-dimensional) models of phytoplankton. The workflow of this framework is demonstrated using two marine algal species, one dinoflagellate *Prorocentrum micans* and one diatom *Halamphora* sp. The resulting 3D models are made openly available and allow users to interact with phytoplankton and their complex structures virtually (digitally) and tangibly (3D printing). They also allow for surface area and biovolume calculations of phytoplankton, as well as the exploration of their light scattering properties, which are both important for ecosystem modeling. Additionally, by presenting these models to the public, it bridges the gap between scientific inquiry and education, promoting broader awareness on the importance of phytoplankton.

## 1 Introduction

Marine phytoplankton, the “invisible forest” in the sea, account for around 50% of the total primary production on Earth (Longhurst et al., 1995; Field et al., 1998), comparable to the terrestrial plants. They also play a substantial role in oxygen production within the ocean (Falkowski, 2012). Highly diverse (Ibarbalz et al., 2019), operating at the base of the marine food web (Michaels and Silver, 1988), phytoplankton provide essential services in climate regulation due to their crucial role in the global carbon cycle (Cermeño et al., 2008). Given their importance in marine ecological and biogeochemical processes, monitoring the composition, distribution and variation of phytoplankton has always been a hotspot for research (Boyce et al., 2010; Righetti et al., 2019).

Unlike plants on the land, phytoplankton are microscopic (0.2–200 μm), single-celled organisms and invisible to the naked eye. At the species level, visually identifying phytoplankton traditionally relies on light microscopy (Tomas, 1997). It is relatively simple to use and widely available, producing information on phytoplankton morphology, such as their size, shape and structure. However, the magnification capabilities of light microscopes are limited, making it difficult to observe small features of phytoplankton cells. Scanning electron microscopy (SEM) has higher magnification and resolution than light microscopy. This allows for detailed observations of small features of phytoplankton cells (e.g., external structure and surface ornamentation), extending the capabilities for microscopic identification (Pearl and Shimp, 1973). SEM images of phytoplankton have been used for various purposes including identifying and classifying new phytoplankton species (Han et al., 2016; Li et al., 2017), distinguishing between toxic and non-toxic species (Lim et al., 2012), exploring phytoplankton functions (Iverson et al., 1989; Uwizeye et al., 2021), investigating the impact of environmental factors on phytoplankton cell morphology (e.g., ocean acidification, Cubillos et al., 2007; Bach et al., 2011), and monitoring phytoplankton populations and composition (Yoshida et al., 2020).

Although SEM has helped improve understanding on the morphology of phytoplankton, it remains difficult to convey the detailed and complex structure of phytoplankton through their 2D (two-dimensional) images. With the development of 3D (three-dimensional) printing technology and the increasing accessibility of 3D printers (Jones, 2012), it is now easy to scale and replicate 3D models of microscopic phytoplankton to over 1,000 times their original size, allowing actual morphological features of phytoplankton to be observed by eye without a microscope. Structure-from-motion (SfM) photogrammetry is a technique used to derive 3D information from overlapping 2D images (Remondino and El-Hakim, 2006). It has been applied widely to create 3D models using multiple photographs of an object or scene in various fields of research (e.g., archaeology, geomorphology, geology, agriculture, Reu et al., 2013; Mosbrucker et al., 2017; Squelch, 2017; Gil-Docampo et al., 2019). Combining the principle of SfM with the advantage of SEM photogrammetry enables the creation of 3D models of ultra-small objects (Andrade et al., 2015; Gontard et al., 2016; Ball et al., 2017). This can be done using a series of 2D SEM images of different views of the object taken by rotating the stage during SEM observation. Using low-cost or open-source image-based modeling software, the digital reconstruction of a 3D model can be achieved automatically and precisely (Eulitz and Reiss, 2015; Kozikowski, 2020; Sihvonen and Reinikainen, 2022).

In this study, we develop a workflow to create 3D models for two phytoplankton species (i.e., *Prorocentrum micans* and *Halamphora* sp.) by integrating SEM with photogrammetry. We demonstrate how these models can be scaled to human proportions using 3D printing, allowing their morphology to be tangibly revealed, and how the models can be used for studying characteristics of phytoplankton (e.g., surface-to-volume ratios and light scattering properties), useful in ecological and optical modeling. Our 3D models are made publicly available to promote further research and creative education.

## 2 Materials and methods

### 2.1 Specimens

**Figure 1:**
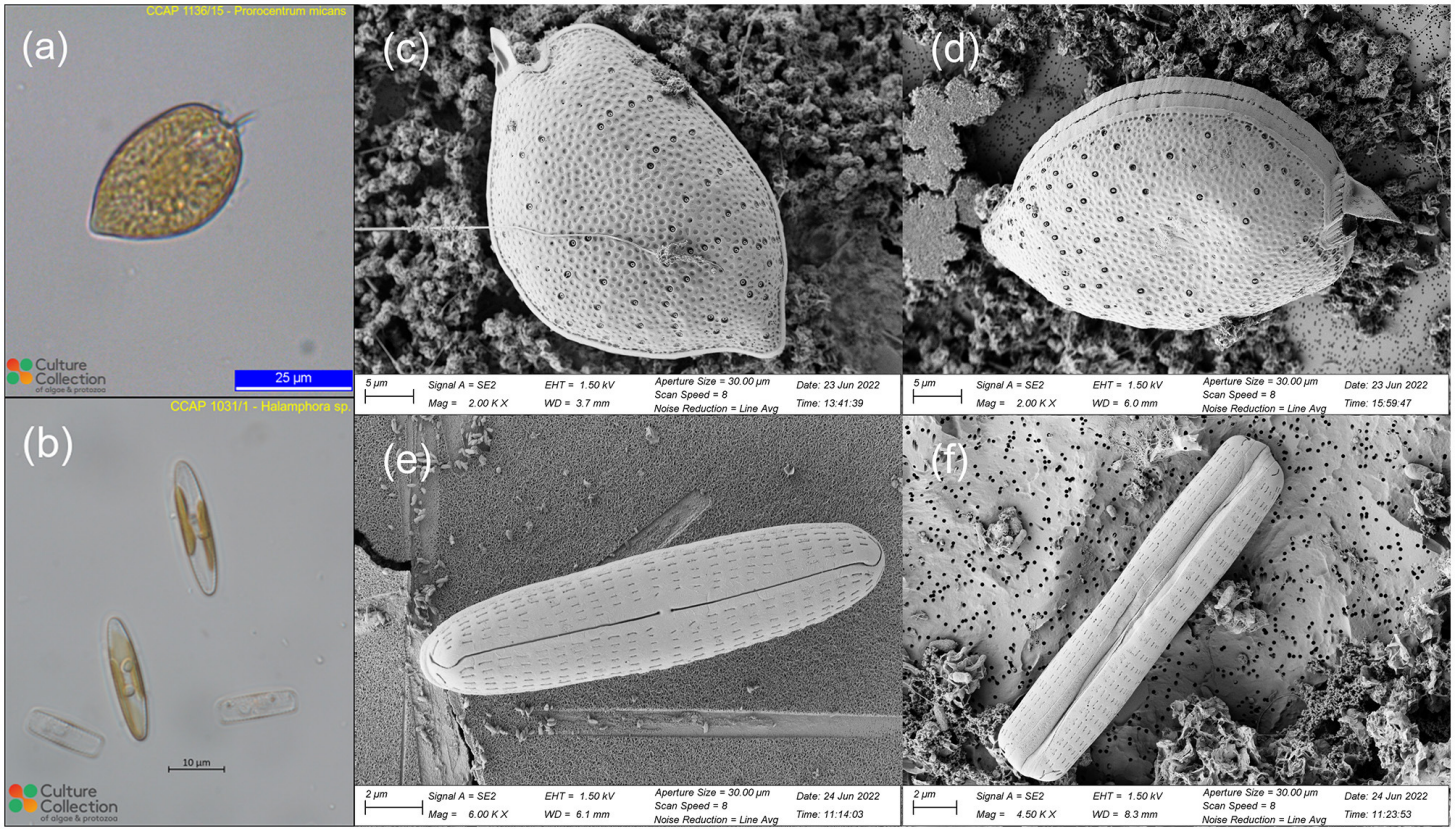
Light microscopic images of *P. micans* **(a)** and *Halamphora* sp. **(b)** used in this study, obtained from the CCAP website (https://www.ccap.ac.uk/catalogue/strain-1136-15 and https://www.ccap.ac.uk/catalogue/strain-1031-1). Scanning electron microscopic images of *P. micans* **(c, d)** and *Halamphora* sp. **(e, f)**, obtained from this study from different viewing angles.

Two live marine uni-algal species were obtained from the Culture Collection of Algae and Protozoa (CCAP, https://www.ccap.ac.uk/): one dinoflagellate *Prorocentrum micans* (CCAP 1136/15), and one diatom *Halamphora* sp. (CCAP 1031/1). The *P. micans* and *Halamphora* sp. were cultured by CCAP in L1 and f/2+Si medium, respectively, at a temperature range of 15–20°C, under a 12-h light/12-h dark cycle of a mix of cool and warm fluorescent illumination with the intensity of 30–40 μmolm^−2^*s*^−1^. The specimens were ordered 3–4 weeks before the SEM experiment to ensure that they are in the exponential growth stage. The light microscopy images of the two specimens from CCAP are shown in Figure 1. The shape of *P. micans* are pyriform, which have a width of ~25 μm and length of 40 μm, with the widest point located at their center (Figure 1a). The cells exhibit a rounded shape at their anterior end and taper to a point at the posterior end, with a short, winged spine that originates from the anterior of the cell. The *Halamphora* sp. are elongated, with a width of ~5 μm and length of 22 μm (Figure 1b). The cells are rectangular when viewed from the girdle and have an oblong shape in valve view. One chloroplast is located on each side of the raphe.

### 2.2 Scanning electron microscope analyses

The scanning electron microscope (SEM) analyses involved fixation, washing, dehydration, drying, mounting, coating, and imaging the cells, in-line with previous studies (Tillmann, 2018; Koon et al., 2019). For ultrastructural analysis, liquid cultures of live uni-algal species were fixed in 2% glutaraldehyde and 2% paraformaldehyde in culture medium (f/2 medium) and kept at 4°C overnight. The fixed samples were then transferred onto polycarbonate filters (Whatman Nuclepore Track-Etched Membranes, 0.2 μm, 25 mm) using a glass filtration rig under low pressure vacuum. After undergoing three rounds of 5-min washes in deionised water to remove fixation reagents and sea salt completely, the samples underwent dehydration via a graded ethanol series (i.e., 30%, 50%, 70%, 80%, 90%, 95%, and 100%), where the first six grades were conducted for 5 min and the last one was subjected to two 10-min rounds. The samples were then incubated in HMDS (hexamethyldisilazane, Merck, Gillingham, UK) for 3 min, followed by air drying. Dried specimens were then mounted on aluminum stubs (G301, Agar Scientific), coated with 10 nm gold-palladium (Q150T sputter coater, Quorum, Lewes, UK), and observed under a scanning electron microscope (GeminiSEM 500, Zeiss) operated at 1.5 kV using a SE2 detector. High-resolution SEM images of the two specimens were saved in Tag Image File Format (TIFF; Figure 1). In contrast to images from light microscopy, the SEM images capture cell ultrastructure morphological features. The surfaces of the valves of *P. micans* are full of depressed ornamentation, accompanied with an abundance of both large and small pores (Figures 1c, d). The large trichocyst pores are densest in the lower valve, arranged in radial rows around the margins. The apical periflagellar region are characterized by a moderately excavated, U-shaped triangular depression, with a strong winged spine and an evident small collar. The valves of *Halamphora* sp. are linear-elliptic in outline with rounded apices (Figures 1e, f). The proximal raphe ends are straight and simple, whereas the distal ones are strongly bent. An uninterrupted uniseriate striae, consisting of lineolate and rectangular areolae, extends from the valve face to the mantle.

### 2.3 Photogrammetric mapping and 3D reconstruction

For *P. micans*, multiple SEM images of the same individual cell from different orientations were captured for the subsequent 3D reconstruction, based on the principles of SfM photogrammetry (Eulitz and Reiss, 2015; Ball et al., 2017, and references therein). In brief, during the imaging process, one algae with healthy cell structure was selected as the subject and kept at the center of the field of view, to ensure that sufficient overlaps among adjacent images can be achieved. Given the small size of the algal cell and the presence of multiple cells in the field of view, tilt and rotation angles were adjusted manually. Eventually, a total of 59 clear images of *P. micans* were captured with different rotation angles at 10 tilt angles (i.e., –15, 0, 10, 15, 20, 25, 30, 35, 40, and 45 degree).

**Figure 2:**
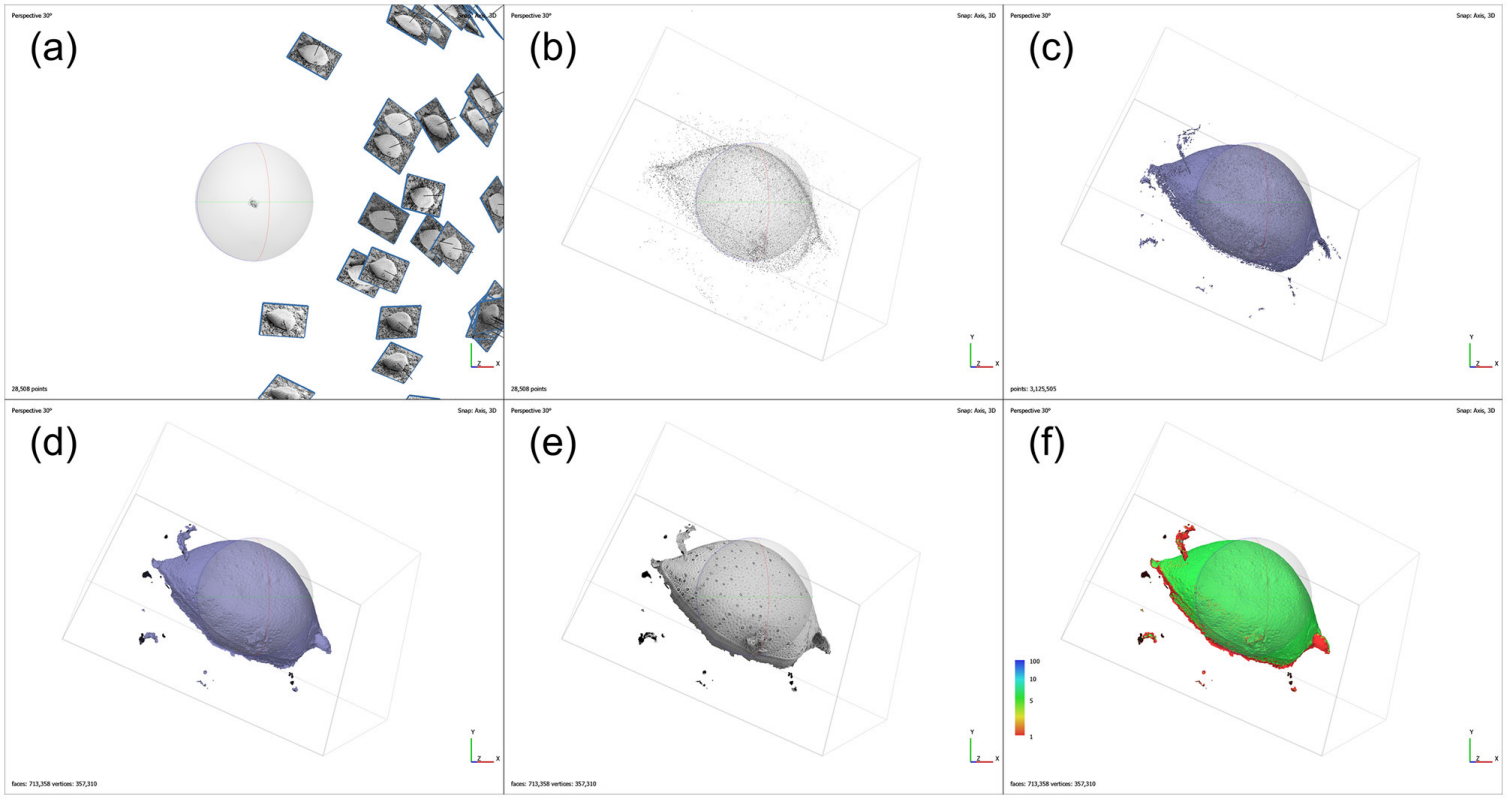
Photogrammetry processing workflow of SEM images of *P. micans* in Agisoft Metashape, including: simulated camera positions from image alignment **(a)**; 3D point cloud **(b)**; 3D dense cloud **(c)**; 3D mesh model **(d)**; 3D textured model **(e)**; and the confidence of the model **(f)**.

The 3D reconstruction was conducted using Agisoft Metashape software (Professional edition, version 1.8.5). After importing 59 SEM images of *P. micans* into the software, the irrelevant elements, such as the background, were masked manually, and the coincident masks of each image were exported and saved for subsequent processing stages. The images were then aligned automatically, with the “High” accuracy setting and respective limits of “40,000” key points and “10,000” tie points. Camera position estimation of each image was carried out during alignment to create a consistent coordinate system for the 3D model (Figure 2a), and tie points were generated accordingly. At this stage, the previously created masks were applied to the tie points. The point cloud generated from the images is shown in Figure 2b. Afterwards, a dense point cloud was generated, with “Ultra High” quality and “Mild” depth filtering settings, as shown in the Figure 2c. The point cloud data was then used to create a mesh consisting of interconnected polygons, with “Dense cloud,” “Arbitrary (3D)” and “High” applied to “Source data,” “Source type” and “Face count,” respectively. The generated mesh model of *P. micans* is shown in the Figure 2d, which was then exported in the OBJ (object file) format for further optimisation. The texture of the model was created based on the SEM images (Figure 2e), which corresponds with the appearance of the algal cell. The confidence of the model (Figure 2f), as calculated in the dense point cloud procedure, reveals that the valve region of the model is more certain than the intercalary band region, which required further modification as described in the following steps.

### 2.4 Printable model visualization

**Figure 3:**
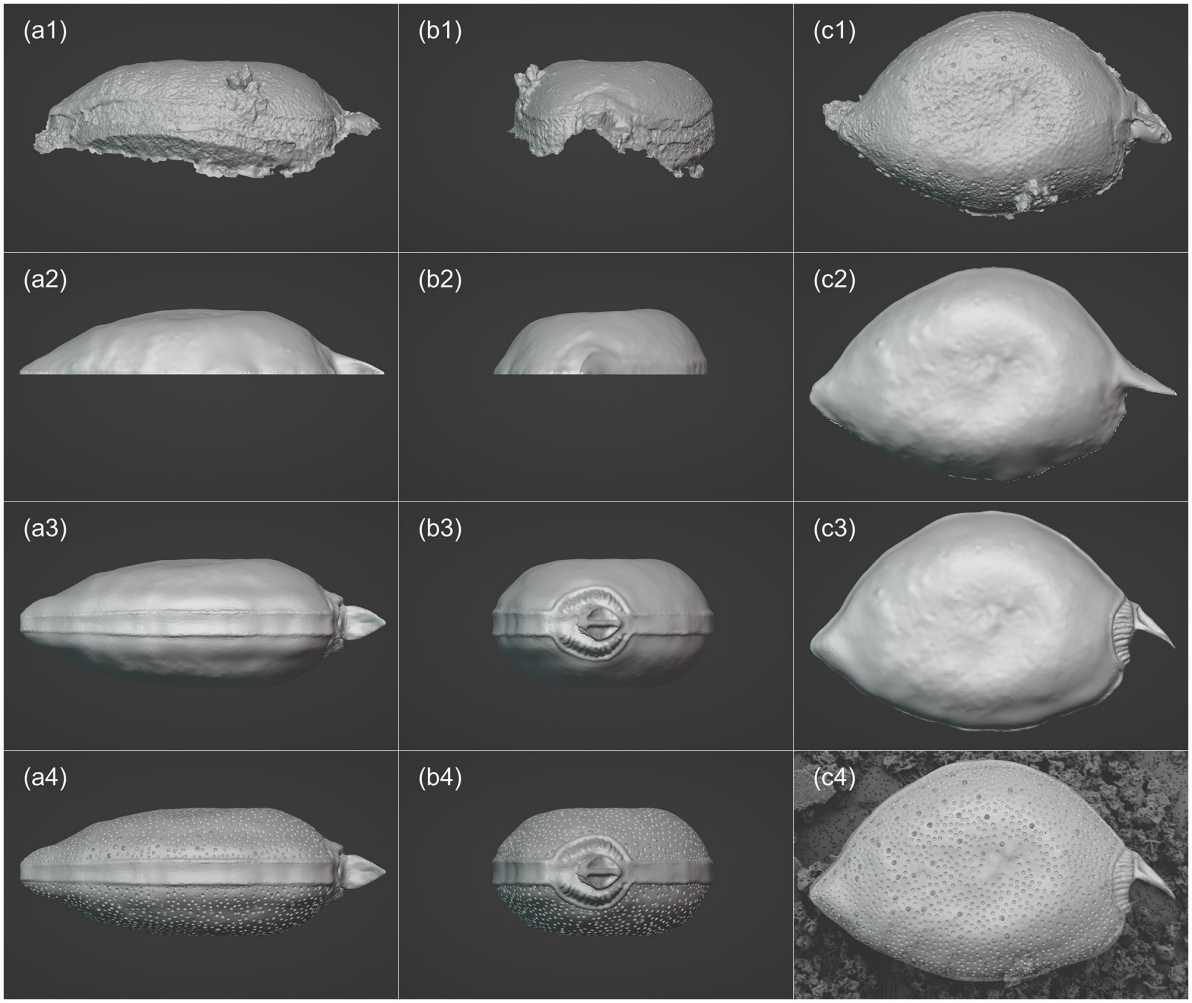
Visualization processing workflow of *P. micans* in Blender, including: importing the base model derived from SEM Images and photogrammetry **(a1–c1)**; adjusting resolution, orientation and angles **(a2–c2)**; mirroring the model and making basic morphology adjustments **(a3–c3)**; enhancing model details **(a4–c4)**, with the background in c4 showing the SEM image overlaid with the 3D model for reference. From left to right: frontal (girdle) view, side (apical) view, and top (valve) view.

3D models of the two phytoplankton were created using the software Blender (version 3.5.1, https://www.blender.org/). The refinement of the 3D model for *P. micans* is based on the previously generated model by Agisoft Metashape (i.e., the OBJ file), as shown in Figures 3a1–c1. Due to the high specification and quality processing steps applied in the Agisoft Metashape, the model was outputted with excellent resolution. To reduce the consumption of memory and time in the following steps, the resolution of the model was reduced using the “Decimate” modifier. It was observed that the cell adhered to the filtration membrane at a non-uniform angle, resulting in the generated model that lacked symmetry along the *XYZ* axes (Figures 3a1, b1). To facilitate subsequent modeling and 3D printing, manual adjustments were made to the orientation. The angles of the imported model was adjusted to align its long axis with the *X*−*Y* plane and be perpendicular to the *Z*-axis. The noise and artifacts of the model (outliers in Figure 2d) were then removed (Figures 3a2–c2). For parts of the cell, i.e., left valve, that was not captured during the SEM analysis and was not constructed through photogrammetry, it required manual completion. Previous studies demonstrated differences between the left and right valves of the *P. micans* (e.g., the number and location of trichocyst pores, periflagellar area, Han et al., 2016; Tillmann et al., 2019). However, only one cell was observed from multiple orientations under SEM in this study, and therefore, these unseen parts were processed under the assumption that the *P. micans* consists of two symmetrical convex valves. The right valve was focused on initially and the left was replicated using the “Mirror” modifier (Figures 3a3–c3). Subsequently, in “Sculpt” mode, the mesh surface of the model was fine-tuned, using SEM images as reference. The valve margins, thecal pores, trichocyst pores, periflagellar area and other morphological features were further modified, formed, shaped, adjusted and smoothed (Figures 3a4–c4) by overlaying the SEM image with the 3D model in Blender (e.g., background in Figure 3c4). Ultimately, the 3D model of *P. micans* was saved and exported in both OBJ and STL (stereolithography) formats.

**Figure 4:**
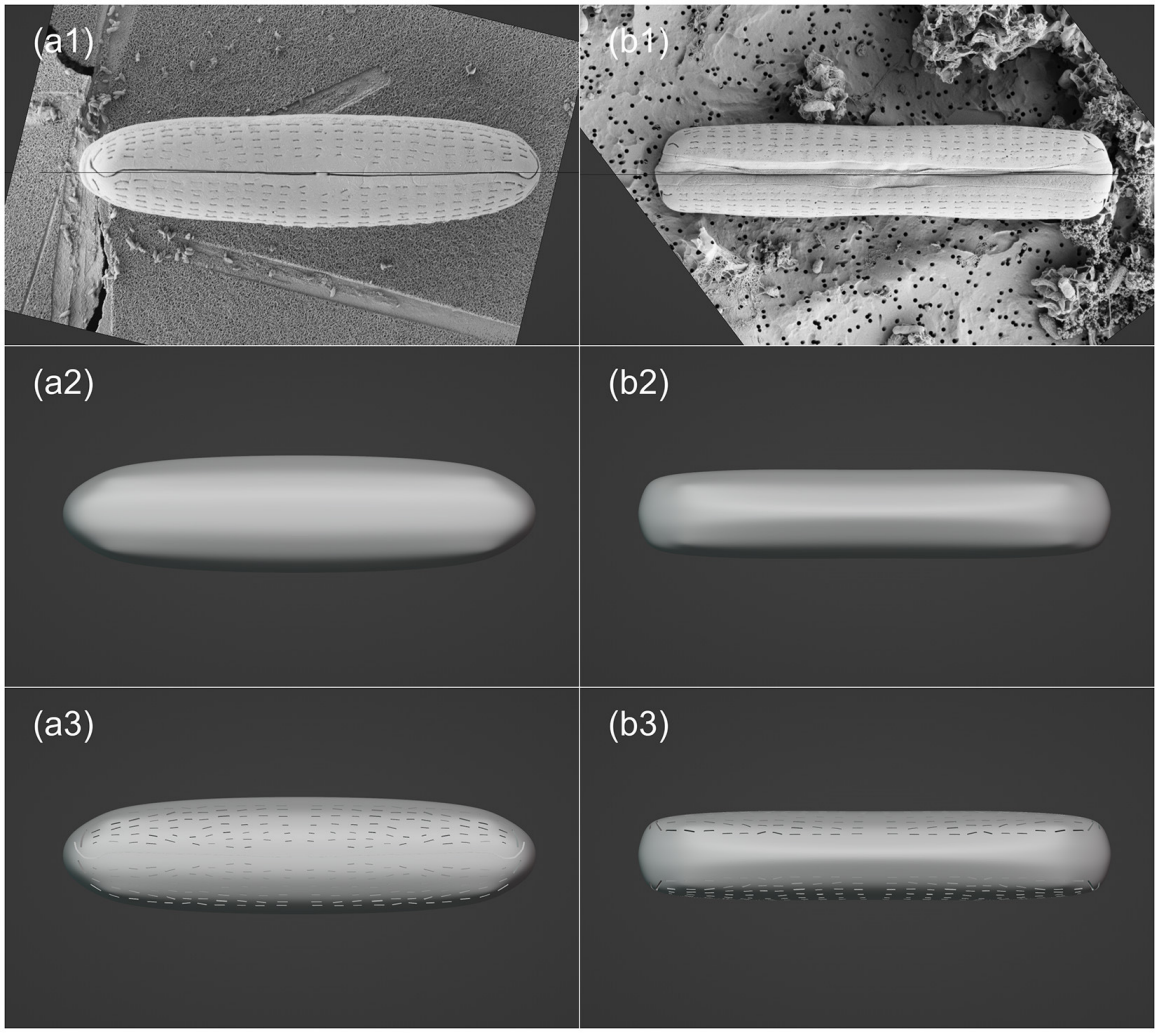
Visualization processing workflow of *Halamphora* sp. in Blender, including: importing SEM images **(a1–b1)**; creating mesh and making basic morphology adjustments **(a2–b2)**; enhancing model details **(a3–b3)**. The left and right columns are top (valve) view and frontal (girdle) view, respectively.

Unlike the photogrammetry-based 3D reconstruction approach applied to the *P. micans*, the 3D model of the *Halamphora* sp. was constructed using SEM images of two individual cells from the front view and the side view, respectively. Firstly, two SEM images were imported into the Blender and their position and angle were adjusted to fit the front and side views (Figures 4a1, b1). A “Round Cube” mesh was then created, with the “Size” and “Divisions” set referring to the two images (Figures 4a2, b2). Assuming that the *Halamphora* sp. is symmetrical in three dimensions, the initial focus was on the 1/8 of the cell, with the “Mirror” modifier being used for the rest. In the “Edit” mode, the vertices, edges, and faces of the mesh were moved and adjusted to generate the basic model shape. The slit-like raphe and areolae on the cell were then extruded using the add-on Boxcutter (version 7.1.9, https://blendermarket.com/products/boxcutter), as shown in Figures 4a3, b3. Finally, the 3D model of *Halamphora* sp. was saved and exported in OBJ and STL formats.

### 2.5 3D printing

**Figure 5:**
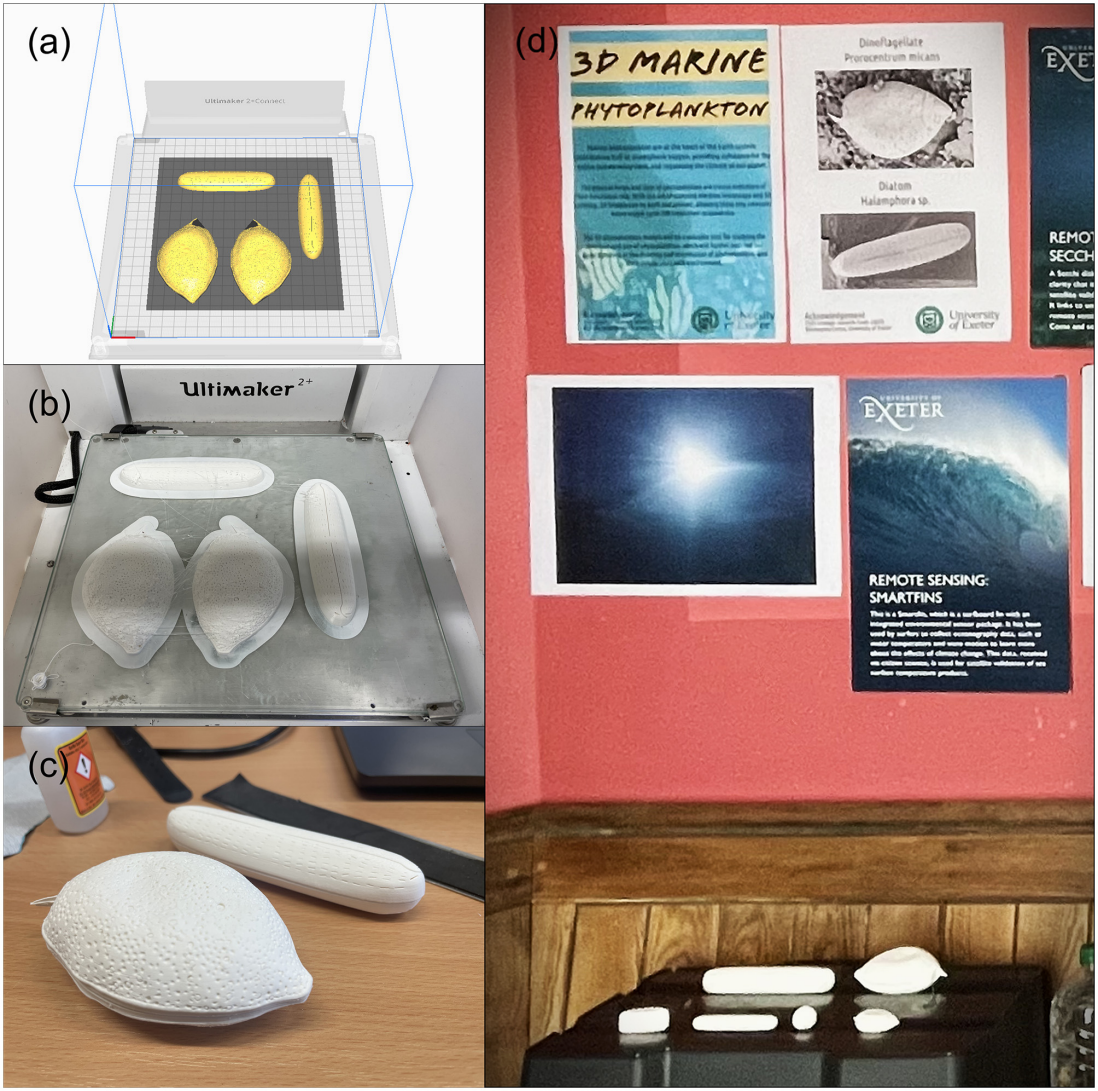
3D printing and 3D models, including: preparation of the 3D printing of the two models using an Ultimaker 3D printer and its software **(a)**; the printed result of the two models **(b)**; assembling the models and presenting the final products **(c)**; and a public exhibition of the models at the Pop-Up Curiosity Shop of Science and Culture (https://futuresnight.co.uk/events/pop-up-curiosity-shop-of-science-and-culture/) **(d)**.

The 3D models of two phytoplankton were printed using the Ultimaker 2+ Connect (https://ultimaker.com/3d-printers/ultimaker-2-plus-connect). Before printing, models were divided into two parts along the *Z*-axis, and were placed on the same horizontal plane to be printed separately. Subsequently, the model was sliced into layers using the software Ultimaker Cura (https://ultimaker.com/software/ultimaker-cura, Figure 5a), where print parameters (e.g., resolution) were set based on the requirements. Once the printer was set up and the model sliced, the 3D printer built the model layer by layer, using the filament to create the structure and shape of the object (Figure 5b). Finally, the two parts of the model were glued together (Figure 5c).

## 3 3D model analysis and applications

### 3.1 3D model reconstruction and comparison

**Figure 6:**
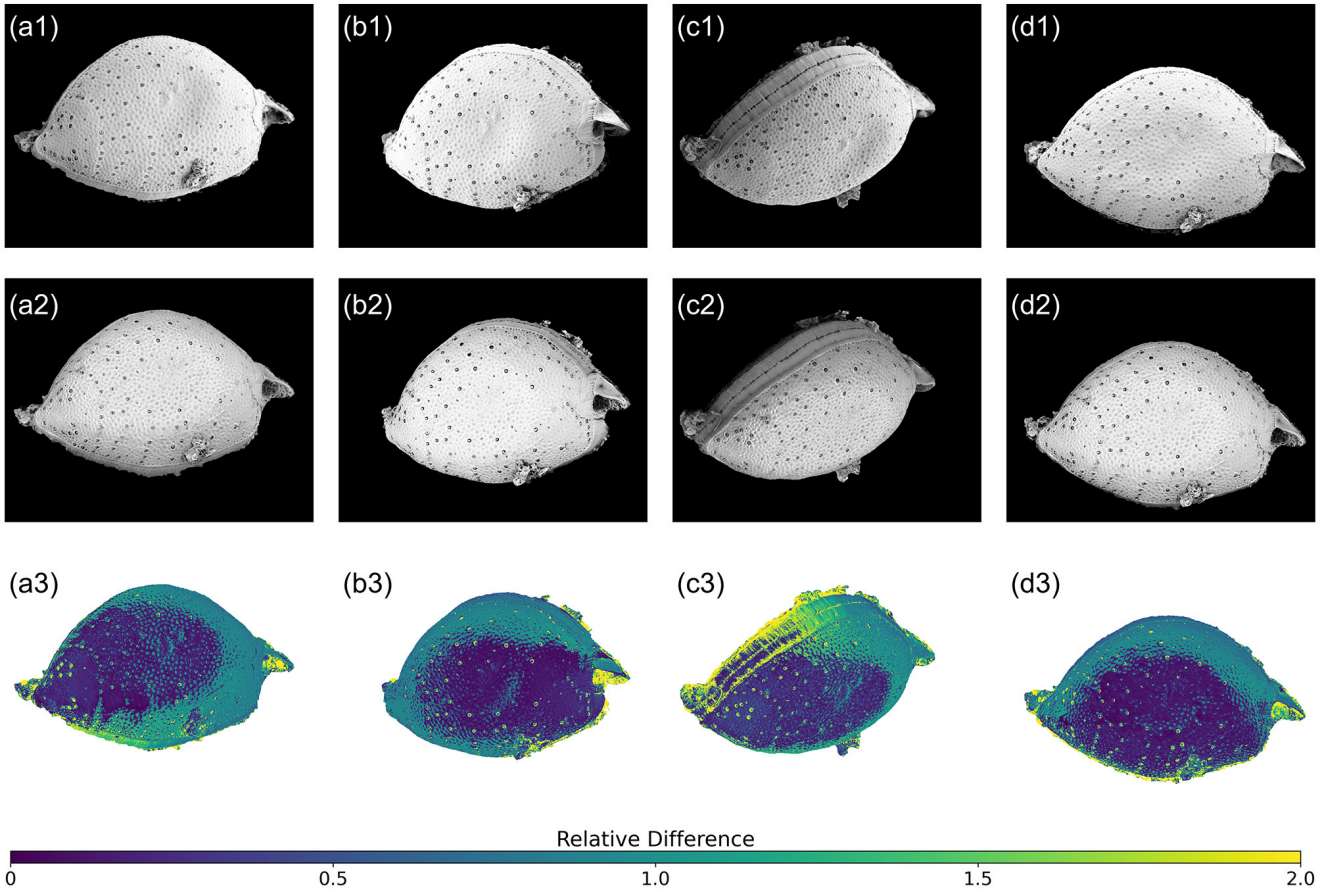
Relative difference between reconstructed 3D model and original SEM images of *P. micans* at different tilt and rotation angles. The first row **(a1–d1)** are the SEM images, the second row **(a2–d2)** are the corresponding 3D model with texture generated through Agisoft Metashape, and the third row (a3–d3) are the relative differences between models and images.

To verify the accuracy of SfM photogrammetry-based 3D model of *P. micans*, we compared the textured model with the original SEM images. Of all the 59 SEM images used for 3D model construction, four images captured from different angles were chosen for a detailed comparison (Figure 6). The textured 3D model generated using the Agisoft (Figure 2e) was firstly paired with the SEM images at the equivalent angles and sizes. Subsequently, the view of the textured 3D model was captured for comparison. Edge detection (“OpenCV” package in Python) was employed to outline the edge of the model automatically, and the resulting mask was applied to both images, as shown by the black background in Figure 6. To mitigate the impact of brightness variations from the two image sources on the comparison, histogram matching (“skimage” package in Python) was performed on the images of the textured 3D model, aligning them with the original SEM images. The relative difference for each pixel in the paired images was then calculated as $|X_{i}^{M}\;-X_{i}^{S}|/X_{i}^{S}$, where $X_{i}^{M}$ and $X_{i}^{S}$ represent the pixel value of the *i*-th pixel in the model and SEM images, respectively.

In general, the relative differences between paired images are affected by the spatial relationship between the sample stage and the lens. Notably, regions closer to the lens show smaller differences, indicated by dark blue colors. This underscores the importance of multiple observations from various rotation and tilt angles to achieve an accurate 3D model reconstruction. Furthermore, higher relative differences are apparent in pores, especially trichocyst pores, spanning the entire cell. These differences are due to elevation differences in these pores, compared to the cell surface. Unlike other image sources, SEM images lack elevation information, posing challenges in 3D reconstruction using SfM photogrammetry. This limitation is evident in Figure 3c1, where morphological details of the pores are absent, replaced by depressions. Consequently, further refinements and adjustments to the model are necessary, as illustrated in Figure 3c4. Additionally, the comparison highlights differences at the edges, which is consistent with the uncertainty calculated directly from the software (Figure 2f). For example, the model struggles to accurately represent the contact surface between the cell and the filter membrane, partly due to impurities (e.g., nutrient salt granules from the seawater medium and excretions from algae growth) on the filter, introducing significant noise in the SEM images. Despite manual masking of non-target areas during software modeling, some impurities remain. Owing to the constraints of the SEM microscope observation angle and distance, few images focusing on these regions were available, contributing to increased uncertainty. Nonetheless, despite differences between the model and the original images, 3D modeling based on SfM photogrammetry provides a convenient foundation for subsequent model refinement and alleviates the difficulties of constructing models from scratch.

### 3.2 Implications for research and education

#### 3.2.1 Surface area and biovolume calculation and assessment

Cell size and morphology is fundamental in various phytoplankton physiological and ecological processes, including nutrient uptake and growth rates (Litchman et al., 2007), light absorption (Finkel, 2001), abundance and biomass (e.g., size-abundance spectrum, Marañón, 2015), and taxonomic community structure (Ryabov et al., 2021). These factors are critical for estimating primary production and carbon export (Siegel et al., 2014). Cell morphology, typically characterized by surface area and biovolume, plays an important role in how efficiently cells can absorb nutrients and light. Numerous methodologies have been employed to calculate surface area and biovolume for phytoplankton, ranging from simplistic geometric models to 2D imaging and realistic 3D models (Hillebrand et al., 1999; Sun and Liu, 2003; Moberg and Sosik, 2012; Roselli et al., 2015; Borics et al., 2021; Mohan et al., 2021).

Table 1 shows the size and morphology information obtained from SEM images for two algal species, including length, width, and depth (or height), derived from the Fiji software (https://imagej.net/software/fiji/). For the *P. micans*, the observations were made from multiple images of the same individual cell used to construct the 3D model, whereas for the *Halamphora* sp., observations were made from different individuals within the field of view. Due to potential uncertainties caused by the angle of observations (Figures 2, 6), measurements were only retained when SEM images were taken at small angles of tilt, with the aim of aligning the plane of observation as parallel as possible to the lens. Consequently, depth information was not measured for *P. micans*. Measurements of the size of the constructed digital 3D models (STL files) of the two algal species, obtained using MeshLab (https://www.meshlab.net/, Cignoni et al., 2008), are listed in Table 1. For the *P. micans*, the 3D model was generated based on the principle of photogrammetry and underwent optimisation adjustments (Sections 2.3 and 2.4), leading to minor differences between the 3D model and the SEM images. Meanwhile, the length, width, and depth information of the *Halamphora* sp. model were derived directly from SEM images, resulting in the same values.

**Table 1:** Morphological characteristics and measurements of algal species derived from SEM imaging and 3D modeling (STL files), with reference to those derived from previous studies (Sun and Liu, 2003; Borics et al., 2021).

***Prorocentrum micans***				
		**Length**	**Width**	**Depth**
SEM images	Mean (μm)	45.10	26.86	–
	STD (μm)	0.69	0.75	–
	*N*	6	6	–
3D model	Mean (μm)	45.10	27.33	15.83
		This study	Sun and Liu (2003)	Borics et al. (2021)
Surface area (μ*m*^2^)		2,341.26	2,597.66	3,046.12
Biovolume (μ*m*^3^)		8,714.20	10,216.35	14,762.77
		This study	Sun and Liu (2003)	Borics et al. (2021)
Diameter for spherical equivalent surface area (μm)		27.30	28.76	31.14
Diameter for spherical equivalent biovolume (μm)		25.53	26.92	30.44
***Halamphora*** **sp**.				
		**Length**	**Width**	**Depth**
SEM images	Mean (μm)	16.27	3.94	3.24
	STD (μm)	2.14	0.24	0.46
	*N*	10	6	4
3D model	Mean (μm)	16.27	3.94	3.24
		This study	Sun and Liu (2003)	Borics et al. (2021)
Surface area (μ*m*^2^)		172.59	203.55	272.59
Biovolume (μ*m*^3^)		137.96	163.12	214.42
		This study	Sun and Liu (2003)	Borics et al. (2021)
Diameter for spherical equivalent surface area (μm)		7.41	8.05	9.31
Diameter for spherical equivalent biovolume (μm)		6.41	6.78	7.43

The surface area and biovolume of the 3D models (STL files) of the two algal species were measured using MeshLab, as shown in Table 1. To compare the results of this study with those obtained from other methods in previous research, the equations of geometric shapes provided in Sun and Liu (2003) and the online generation tool based on 3D meshes from Borics et al. (2021) were used, based on length, width, and depth information of the 3D models. Specifically, *Prorocentrum Ehrenberg* (code 3) in Sun and Liu (2003) and *Characium orissicum* that has a similar drop shape in Borics et al. (2021), were used as references for *P. micans*, while for *Halamphora* sp., the references were *Neidium Pfitzer* (code 11) and *Achnanthidium minutissimum*, respectively. The results indicate that for both algal species, the surface area and biovolume values calculated from 3D models are the smallest, with Sun and Liu (2003) slightly higher and Borics et al. (2021) having the largest values. This trend is also reflected in their respective spherical equivalent diameters. The differences in equivalent diameters based on surface area and biovolume suggest that the shapes of these two algal species are not spherical. In particular, the elongated forms of *Halamphora* sp. show larger differences between spherical equivalent diameters from the surface area and the biovolume. It is important to note that the surface area and biovolume calculations for both algal species in this study were based solely on the results of one single 3D model. Variations in shape or size among different individuals were observed during SEM observation. Therefore, generating multiple models from different individuals of the same species will be necessary in future work to provide a better representation of their variability. Additionally, this study measured the total cell biovolume rather than the cytoplasmic biovolume of phytoplankton. Factors such as cell wall thickness (e.g., silica shells of diatoms) contribute to total cell biovolume, potentially leading to discrepancies when estimating properties like carbon content (Strathmann, 1967). Future studies using techniques like transmission electron microscopy on cell sections could provide more accurate assessments of cytoplasmic biovolume.

#### 3.2.2 Scattering properties

Historically, simulations of the scattering properties of phytoplankton have been based primarily on the assumption that the particles are homogeneous spheres (Bricaud and Morel, 1986; Stramski et al., 2001). However, phytoplankton exhibit a wide range of shapes and morphological complexities. It has been demonstrated that Mie theory for homogeneous spheres cannot accurately predict the scattering properties of irregularly shaped or heterogeneous cells (Volten et al., 1998; Whitmire et al., 2010). Improving our understanding of the impact of phytoplankton shape on scattering properties may help bridge gaps between theoretical assumptions and experimental observations, improving our ability to model radiative transfer processes in the ocean (Stramski et al., 2004).

**Figure 7:**
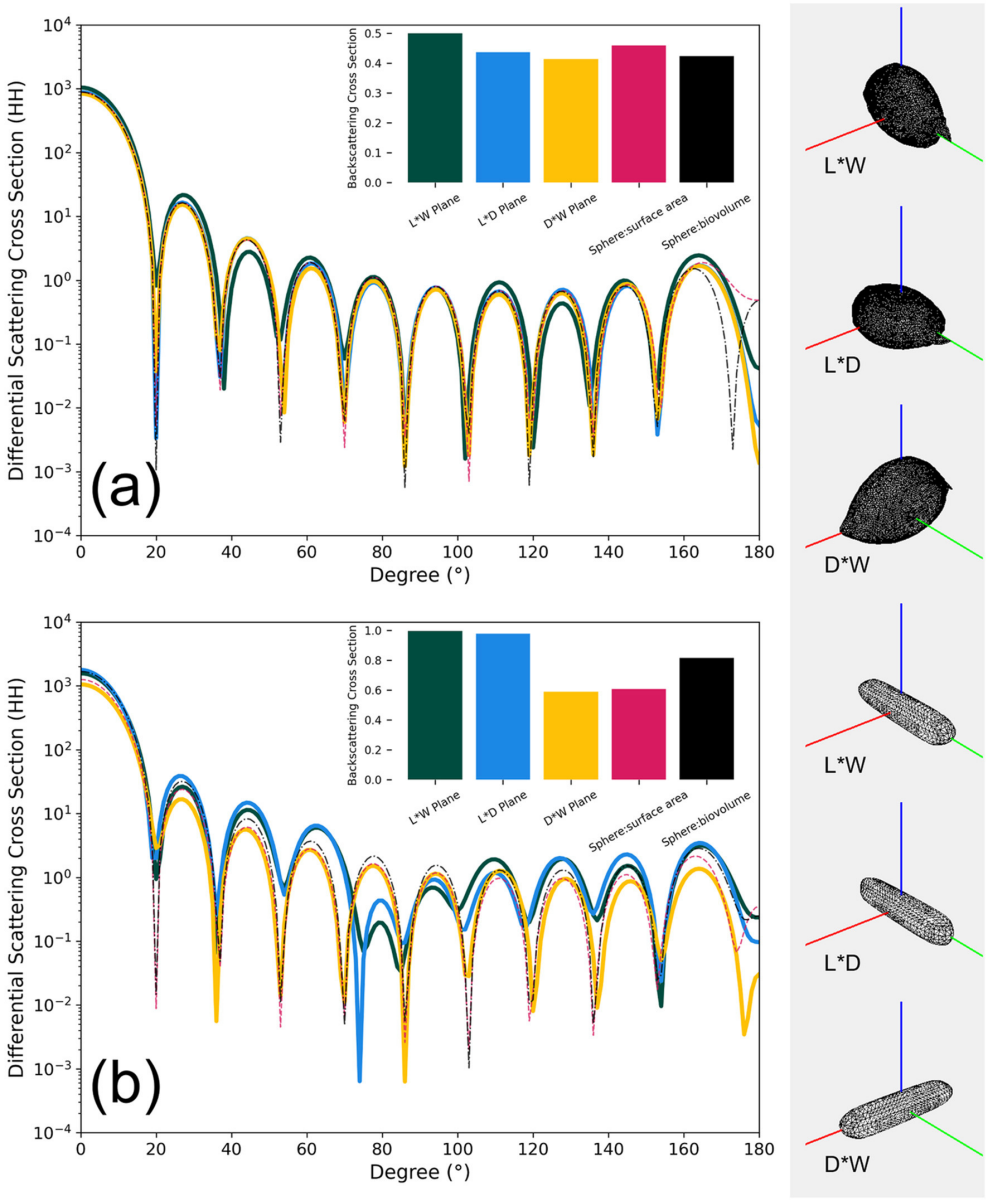
Relationship between the differential scattering cross sections (HH, parallel direction) and angles between different models for two algal species, *P. micans* **(a)** and *Halamphora* sp. **(b)**, at a wavelength of 532 nm. The bar plot within the figure is the respective integral backscattering cross section result. The planes L*W, L*D, and D*W represent length-width, length-depth, and depth-width orientations of the model relative to the incident light direction (represented here by the red line on the left), respectively.

Figure 7 shows the differential scattering cross sections (DSCS) of the two phytoplankton 3D models (OBJ files) under the direction of incident light in the range of 0–180 degrees and from three angles (length-width, length-depth, and depth-width planes). Additionally, spherical 3D models (OBJ files, with diameters derived from equivalent surface area and biovolume, see Table 1) are included for comparison. The DSCS were computed using the Superellipsoid Scattering Tool (SScaTT, v.1.1.4, Wriedt, 2002), based on the T-matrix method. The parameters used in SScaTT were set as follows: a wavelength of 532 nm; the refractive index values representative of phytoplankton were set to a real part of 1.05 and an imaginary part of 0.01 (Clavano et al., 2007); other parameters including radius for normalization, Nrank, and Mrank were set to 0.1, 10, and 7, respectively, following the reference (default) settings (Wriedt, 2002).

*P. micans* has a relatively symmetrical shape, and the differences in DSCS among models are small, with integrated backscattering cross section showing consistent results (Figure 7a). In contrast, for the elongated *Halamphora* sp., significant differences in DSCS values are observed between our constructed 3D models and equivalent diameter-based models (Figure 7b). The shape of the DSCS with respect to angle suggests that the results from the length-width and length-depth planes are more similar, whereas those from the depth-width plane are more consistent with the two spherical models. This consistency is also evident in the results of backscattering integration (Figure 7b), and aligns with the fact that the depth-to-width ratio of *Halamphora* sp. is closer to that of the spherical models. Figure 7b illustrates the importance of accounting for the complex structure and non-symmetric shape of phytoplankton when calculating their scattering properties (Gordon and Du, 2001). However, it is important to note that in addition to shape, other factors not explicitly addressed in our simulations, such as the cell heterogeneity caused by external and internal cellular structures, also play an important role in scattering (Poulin et al., 2018). For example, using homogeneous models, as opposed to heterogeneous models that account for the influence of cell structures, may introduce a relative bias of 0.821 in backscattering coefficients (Organelli et al., 2018).

#### 3.2.3 Education and outreach

In recent years, 3D printing and open-access STL files have facilitated the creation of high-quality scientific 3D models, enriching educational experiences in various fields and promoting practical exploration of complex scientific concepts. 3D printing technology offers a wide range of applications in education, such as replicating inaccessible real-world objects (e.g., planetary landscapes, Horowitz and Schultz, 2014), replenishing scarce resources (e.g., anatomical material, McMenamin et al., 2014), and creating objects that are unavailable in the physical world (e.g., chemical structures, Scalfani and Vaid, 2014). 3D printing benefits education by fostering creativity and design skills and improving student engagement, particularly in STEM (Science, Technology, Engineering, and Math) subjects (Bonorden and Papenbrock, 2022). Additionally, 3D printed models facilitate multisensory learning and are beneficial in special education (Buehler et al., 2014).

Our 3D phytoplankton models could serve as valuable educational resources. They are affordable and reproducible, making them an attractive option for educational institutions or research facilities that lack access to live specimens. Interactive engagement with 3D phytoplankton models like ours may increase public awareness of phytoplankton, shining a light on their diversity and ecological significance (Figure 5d), and helping to facilitate public discussion on topics like climate change, marine pollution, and biodiversity conservation.

## 4 Reflections and recommendations

One of the most important factors impacting the production of 3D models is the data source (Remondino and El-Hakim, 2006), which in this study is SEM images. We encountered some issues and difficulties during the process of sample preparation and SEM image data collection, while also identifying areas for improvement. Firstly, in selecting phytoplankton samples, laboratory-cultured uni-algal specimens were chosen over field-collected ones to ensure sample purity. However, the use of culture medium (f/2 medium) during dilution introduced impurities, impacting the background of the SEM images (see Figure 1) that required time-consuming manual masking (Section 2.3) as opposed to rapid automatic masking (Higueras et al., 2021). Other sample preparation methods were explored, including air-drying (Li et al., 2017). However, in the case of marine phytoplankton samples, air-drying led to the precipitation of salt particles, affecting SEM observations, thus this method was not used. Cryo-SEM is another technique for observing phytoplankton, offering advantages in preserving the near-native ultrastructure of phytoplankton (Kumar et al., 2020). However, it was not used in this study, due to the requirement of a cold stage, which limits the sample rotation necessary for photogrammetry.

Based on the same preparation methods used in this study for *P. micans* and *Halamphora* sp., no effective sample images were observed under the SEM for two other phytoplankton species (i.e., *Emiliania huxleyi* and *Skeletonema* sp.). This might be due to the loss of cells during washing or dehydration, meaning further adjustments may be required when preparing these species for the SEM. During SEM observation, locating the same individual was time-consuming, due to their small sizes. However, we found that certain reference points or textured background could facilitate rapid focusing on the target individual. For example, the prominent scratches on the filter pad near the *P. micans* cell selected in our study served as useful reference points. However, for the *Halamphora* sp., we found SfM photogrammetry methods to be challenging on individual cells due to their high sample density. To overcome this, we sampled different individuals from various angles. Attention should be paid in the future to reducing the phytoplankton abundance (density) in samples when making targeted SEM observations of individual cells.

To enhance the effectiveness of SfM photogrammetry, it is crucial to capture images from multiple angles to ensure comprehensive coverage (Sihvonen and Reinikainen, 2022). However, the SEM holder has limitations in its ability to tilt beyond certain degrees (Section 2.3). Therefore, for capturing samples from high tilt angles, we used 45/90 degree multi stub holder during SEM imaging. In addition, small rotation angles may not be sufficient to generate a reliable 3D surface from 2D images. Therefore, it was necessary to manually adjust the angle of the sample on the holder to achieve maximum coverage and ensure complete image capture. In the process of 3D model construction, a thorough understanding of phytoplankton morphology and structure is essential, as well as selecting appropriate modeling software and tools. To facilitate subsequent optimisation and processing speed, it is recommended to segment the model into appropriate parts and apply the modifiers (Section 2.4) (Adamczak et al., 2019).

The methodology proposed in this study for constructing and printing 3D phytoplankton models does have some limitations. One significant barrier is the lack of publicly available resources for 3D models or SEM images of phytoplankton, particularly given the significant diversity of phytoplankton species (Spaulding et al., 2021). The application of our method requires the preparation of algae samples by cultivation, purchase or in-situ sampling. A comprehensive experimental setup is required, including access to SEM along with expertise in specialized knowledge areas such as algal specimen preparation for SEM observation. Mastering the 3D modeling software requires considerable effort (Mohan et al., 2021). SEM experiments are costly and creating 3D models using the SfM photogrammetry often requires commercial software support. It should be noted that the approach outlined in this study represents only one of many pathways. There are alternative software programs available, some of which are freely accessible. For example, Meshroom (https://alicevision.org/) can be used to generate similar photogrammetry-based 3D models as those produced by Agisoft Metashape in this study. Additionally, emerging image-based modeling techniques that incorporate machine learning and artificial intelligence show promise for future improvements and refinements in this field (Han et al., 2021).

## 5 Summary

We provide a framework for creating 3D phytoplankton models for use in both scientific research and public outreach, by integrating photogrammetry with scanning electron microscopy. We detail what we did to construct and optimize 3D models of two phytoplankton species, *Prorocentrum micans* and *Halamphora* sp., highlighting the challenges we encountered. We discuss how 3D printed phytoplankton models can improve public understanding of phytoplankton diversity and ecological significance, as well how they can be used to advance knowledge in surface area and biovolume calculations and phytoplankton scattering properties. Our 3D phytoplankton models are made openly available to promote further research and scientific applications (Sun et al., 2024).

## Data availability statement

The datasets presented in this study can be found in online repositories. The names of the repository/repositories and accession number(s) can be found at: doi: 10.6084/m9.figshare.c.7220913.

## Author contributions

XS: Conceptualization, Data curation, Formal analysis, Funding acquisition, Investigation, Methodology, Project administration, Resources, Software, Supervision, Validation, Visualization, Writing – original draft, Writing – review & editing. RB: Conceptualization, Funding acquisition, Resources, Software, Supervision, Writing – review & editing. CH: Investigation, Methodology, Resources, Software, Writing – review & editing. JV: Writing – review & editing. ML: Methodology, Software, Writing – review & editing.
